# Primary Metabolite Responses to Oxidative Stress in Early-Senescing and Paraquat Resistant *Arabidopsis thaliana rcd1 (Radical-Induced Cell Death1)*

**DOI:** 10.3389/fpls.2020.00194

**Published:** 2020-02-28

**Authors:** Nina Sipari, Jenna Lihavainen, Alexey Shapiguzov, Jaakko Kangasjärvi, Markku Keinänen

**Affiliations:** ^1^ Viikki Metabolomics Unit, Faculty of Biological and Environmental Sciences, University of Helsinki, Helsinki, Finland; ^2^ Department of Environmental and Biological Sciences, University of Eastern Finland, Joensuu, Finland; ^3^ Department of Plant Physiology, Umeå University, Umeå, Sweden; ^4^ Organismal and Evolutionary Biology Research Programme, Faculty of Biological and Environmental Sciences, University of Helsinki, Helsinki, Finland; ^5^ Institute of Plant Physiology, Russian Academy of Sciences, Moscow, Russia

**Keywords:** *Arabidopsis thaliana*, RCD1, paraquat, methyl viologen, metabolite profiling, oxidative stress, senescence, glycolysis

## Abstract

*Rcd1* (*radical-induced cell death1*) is an *Arabidopsis thaliana* mutant, which exhibits high tolerance to paraquat [methyl viologen (MV)], herbicide that interrupts photosynthetic electron transport chain causing the formation of superoxide and inhibiting NADPH production in the chloroplast. To understand the biochemical mechanisms of MV-resistance and the role of RCD1 in oxidative stress responses, we performed metabolite profiling of wild type (Col-0) and *rcd1* plants in light, after MV exposure and after prolonged darkness. The function of RCD1 has been extensively studied at transcriptomic and biochemical level, but comprehensive metabolite profiling of *rcd1* mutant has not been conducted until now. The mutant plants exhibited very different metabolic features from the wild type under light conditions implying enhanced glycolytic activity, altered nitrogen and nucleotide metabolism. In light conditions, superoxide production was elevated in *rcd1*, but no metabolic markers of oxidative stress were detected. Elevated senescence-associated metabolite marker levels in *rcd1* at early developmental stage were in line with its early-senescing phenotype and possible mitochondrial dysfunction. After MV exposure, a marked decline in the levels of glycolytic and TCA cycle intermediates in Col-0 suggested severe plastidic oxidative stress and inhibition of photosynthesis and respiration, whereas in *rcd1* the results indicated sustained photosynthesis and respiration and induction of energy salvaging pathways. The accumulation of oxidative stress markers in both plant lines indicated that MV-resistance in *rcd1* derived from the altered regulation of cellular metabolism and not from the restricted delivery of MV into the cells or chloroplasts. Considering the evidence from metabolomic, transcriptomic and biochemical studies, we propose that RCD1 has a negative effect on reductive metabolism and rerouting of the energy production pathways. Thus, the altered, highly active reductive metabolism, energy salvaging pathways and redox transfer between cellular compartments in *rcd1* could be sufficient to avoid the negative effects of MV-induced toxicity.

## Introduction

Reactive oxygen species (ROS) such as superoxide (O_2_^•-^) and hydrogen peroxide (H_2_O_2_) are important signaling molecules inevitably formed in aerobic energy metabolism. Although basal levels of ROS are required for normal plant performance and development, in excess they cause oxidative stress that could damage cells and trigger physiological and programmed metabolic pathways, which can induce cell death (Noctor et al., 2015; Mittler, 2017; Waszczak et al., 2018). Plants have developed antioxidant systems and complex signaling networks to maintain redox homeostasis and energy metabolism, and to integrate ROS signals with other cellular signals. Despite the high capacity and redundancy of the ROS scavenging systems, alterations in ROS levels have potentially wide-ranging consequences for metabolic processes, including rearrangements in central metabolic pathways and energy metabolism (Baxter et al., 2007; Scarpeci and Valle, 2008; Lehmann et al., 2009; Noctor et al., 2015; Mittler, 2017).

Typically, exposure to excessive levels of ROS causes the down-regulation of anabolic metabolism (e.g. Calvin cycle) while favoring catabolic metabolism, such as oxidative pentose phosphate pathway (OPPP) and lipid, protein and starch degradation, to provide substrates for the production of ATP and reducing power in the form of NAD(P)H. In *Arabidopsis,* changes in gene expression and sugar levels indicated altered metabolism in response to treatment with ROS-generating herbicide, methyl viologen (MV), and the responses resembled transcriptomic changes in plants adapted to darkness (Scarpeci and Valle, 2008).

Soluble sugars play a central role in energy metabolism and signaling, but also a multifaceted role in respect to ROS (Couée et al., 2006). Soluble sugars can be directed to OPPP for NADPH production, which can contribute to ROS scavenging, or they can be involved in ROS-producing metabolic pathways. NADPH has also a dual role in ROS homeostasis, because it serves as a donor of reducing power for ROS processing and facilitates apoplastic ROS generation by the plasma membrane NADPH oxidases. Furthermore, NADP(H), as well as NAD(H), essentially link metabolism to redox signaling, and alterations in their concentrations and redox states strongly affect metabolic pathways involved in ROS responses (Munné-Bosch et al., 2013; Noctor and Mhamdi, 2017).

Paraquat (MV) is widely used for weed control and as a tool in plant science as an electron acceptor of Photosystem I (PSI) and inducer of ROS generation. In chloroplast, MV inhibits photosynthesis by attracting electrons from PSI, which in turn inhibits the reduction of ferredoxin and the production of NADPH (Calderbank, 1968; Farrington et al., 1973). At the same time, superoxide (O_2_^•-^) is formed from O_2_ in the MV redox cycle while NADPH is consumed (Cochemé and Murphy, 2009). Chloroplastic superoxide dismutase (SOD) converts superoxide to H_2_O_2_, which is further scavenged by antioxidant system or translocated to other cell compartments (Asada, 1994; Foyer et al., 1994). Without photosynthetic activity, as in plants in darkness or in yeast or animal cells, MV induces the production of ROS in mitochondria (Lambert and Bondy, 1989; Bowler et al., 1991; Taylor et al., 2002; Cui et al., 2019). In yeast and animal cells, MV attracts electrons from various mitochondrial enzymes (e.g. NADPH dehydrogenases) and complexes I and III (Lambert and Bondy, 1989; Cochemé and Murphy, 2008), but the mitochondrial targets of MV in plant cells have not been characterized.

Several mechanisms have been proposed to account for MV-resistance in higher plants, including sequestration of MV, detoxification of ROS by enzymatic antioxidants (Fuerst and Vaughn, 1990; Hart and Di Tomaso, 1994; Tsugane et al., 1999; Chen et al., 2009; Xi et al., 2012; Li et al., 2013), reduced poly(ADP-ribose)polymerase (PARP) activity and/or increased NADH levels (De Block et al., 2005; Ishikawa et al., 2009; Ogawa et al., 2009). Polyamines and their transporters have been proposed to have a role in MV-resistance due to the structural similarities of the herbicide and polyamines and the non-specific transport of MV into vacuoles (Benavides et al., 2000; Fujita et al., 2012; Li et al., 2013; Fujita and Shinozaki, 2014).

*Arabidopsis thaliana rcd1 (radical-induced cell death1*) shows high resistance to MV-induced chloroplastic ROS but is sensitive to ozone and apoplastic superoxide (Ahlfors et al., 2004; Fujibe et al., 2004; Katiyar-Agarwal et al., 2006). RCD1 is a PARP-like protein, belonging to the SRO-gene family (Similar-to-RCD1), yet it has no direct PARP-activity (Jaspers et al., 2009). However, RCD1 has been suggested to be a candidate target for PARP-inhibitors, as SRO-proteins possess a PARP-like domain and are involved in stress responses similarly to PARP-proteins (Rissel et al., 2017). RCD1 has been implicated in redox signaling from both chloroplasts (Fujibe et al., 2004; Hiltscher et al., 2014; Cui et al., 2019; Shapiguzov et al., 2019) and mitochondria (Brosché et al., 2014; Cui et al., 2019; Shapiguzov et al., 2019). RCD1 interacts with several transcription factors that are involved in developmental processes or plant stress responses (Jaspers et al., 2009). RCD1 negatively regulates ANAC013 and ANAC017, which positively regulates mitochondrial dysfunction stimulon genes (Shapiguzov et al., 2019). The MV tolerance in *rcd1* has been previously associated with altered redox status, the high expression of plastidic SOD and ascorbate peroxidase (APX) (Fujibe et al., 2004) and the higher expression of *AOX* genes (Brosché et al., 2014). Nevertheless, no unambiguous cause for MV tolerance in *rcd1* has been found.

The alterations in redox status and metabolite exchange between organelles (e.g. redox valves, redox-regulated transporters) coordinate cellular functions during stress and developmental stages. There are two main redox valves in photosynthetic plant cells, the chloroplastic malate valve driven by photosynthetically produced NADPH that increases subcellular (in mitochondria, peroxisomes, cytosol, and plastids) NADH/NAD^+^ ratios (Krömer and Scheibe, 1996; Selinski and Scheibe, 2019), and the mitochondrial citrate valve, driven by increased reduction level in mitochondria, that reduces subcellular NADP pools (Igamberdiev and Gardeström, 2003). In addition, the mitochondrial malate-aspartate shuttle transfers reducing equivalents from cytoplasm to mitochondria while coupling the TCA cycle to nitrogen assimilation by interconversion and shuttling of oxaloacetate (OAA), aspartate (Asp), glutamate (Glu), α-ketoglutarate (α-KG) and malate. In plants, the TCA cycle is also tightly connected to the GABA shunt, which is the main producer of succinate and which can bypass two steps of the TCA cycle. The catabolism of GABA in mitochondria is also linked to the interconversion of pyruvate (Pyr) and α-KG to alanine (Ala) and Glu (Studart-Guimarães et al., 2007; Fait et al., 2008).

Various TCA cycle organic acids (di- and tricarboxylic acids) and dicarboxylic amino acids (Asp, Glu) are also transported between organelles by di/tricarboxylate transporters *via* a counter-exchange mechanism (Linka and Weber, 2010; Facchinelli and Weber, 2011) α-KG is transported to the chloroplasts *via* 2-OG/malate transporter for ammonium assimilation in the GS/GOGAT cycle, but its enzymatic origin may vary. α-KG is most likely provided by cytosolic and mitochondrial isocitrate dehydrogenases (ICDH's) and aspartate aminotransferases (AAT's). NAD-dependent ICDH are only found in mitochondria, but NADP-dependent isoforms of ICDH are localized in cytosol, mitochondria, peroxisomes or chloroplasts (Hodges, 2002; Foyer et al., 2011). The conversion of isocitrate to α-KG by NADP-ICDH not only produces NADPH, but also functions as a crossroads of shuttling carbon (and reducing power) between organelles, cross-linking metabolic processes (TCA, amino acid biosynthesis, nitrogen assimilation, fatty acid synthesis and energy production). Although ICDH is also found in the plastids, the site of the GS/GOGAT pathway, the cytosolic ICDH is reported to play a major role (90%) in α-KG production for amino acid synthesis and to be predominant isoform in plants in control growth conditions (Mhamdi et al., 2010; review by Foyer et al., 2011). This is in line with previous studies of TCA cycle to function only partially in light while mitochondrial citrate (precursor for α-KG) is exported to cytosol (and/or other organelles) for amino acid assimilation. However, the loss of cytosolic NADP-ICDH activity did not have a large impact on leaf compounds associated to C and N metabolism, indicating fine-tuned redundancies between the isoforms localized in other cell organelles (Foyer et al., 2011; Mhamdi and Noctor, 2015). The activation of citrate efflux from mitochondria to cytosol decreases the carbon flow to the rest of the TCA cycle leading to reduced levels of mainly malate and fumarate due to redox and thioredoxin regulation (Daloso et al., 2015).

To study the function of RCD1 in plant energy metabolism, untargeted metabolite profiling was performed to assess the metabolic features of *rcd1* mutant in light, in response to MV exposure and after extended darkness. The primary metabolite responses of MV-resistant (*rcd1*) and MV-sensitive (Col-0) plant lines were compared to elucidate the tolerance mechanisms in the mutant plants. The possible functions of RCD1 in the regulation of cellular redox status, and central carbon and nitrogen metabolism are discussed.

## Materials and Methods

### Plant Material

*A. thaliana* wild type (Col*-*0) and *rcd1-4* (GK-229D11, Col-0 background) seedlings were grown on 1× MS with 0.5% Phytagel without sucrose on plates (metabolomics) or on multi-well plates (histochemical staining). Plants were grown under 12-h photoperiod in 150 µmol m^-2^ s^-1^ light for 14 days. Prior to the dark period (12 h night), 5 ml (plates) or 0.5 ml (wells) of MQ water (control) or MV (50 µM) was added. The seedlings were kept in the growth room conditions either for 16 h in darkness (for the dark treatment), or exposed to 12 h of darkness overnight followed by 4 h of growth light in the morning (for light and MV treatments). Seedlings for metabolite analysis were harvested, and pooled plant samples (approx. 100 mg) were snap-frozen in liquid nitrogen and stored at -80°C. Seedlings were pooled due to small size and to ensure detectability of low concentration metabolites. Experiment was performed once with 2-h light exposure time and twice with 4-h light exposure, in presence of MV, or extended darkness. The results from all three experiments were consistent, even though plants showed more intensive responses with 4-h exposures and data from one 4-h experiment is shown.

### Metabolite Extraction and Derivatization

Primary metabolites were analyzed with gas chromatography–mass spectrometry (GC-MS) according to Roessner et al. (2000) and starch was analyzed from the plant residue after extraction of soluble metabolites with the method described in Smith and Zeeman (2006). GC-MS analysis was executed for 6–8 pooled plant samples. Plant material was homogenized with a ball mill (TissueLyser II, Qiagen, Germany) with 1–1.5 mm glass beads. Powdered plant material was extracted twice, first with 1 ml of 100% methanol (Merck) and then with 80% (v/v) aqueous methanol. Internal standards (benzoic-d_5_ acid, glycerol-d_8_, 4-methylumbelliferone) were added to each sample during the first extraction step. During both extraction steps, samples were vortexed for 30 min and centrifuged for 5 min at 13,000 rpm (13,500×g) at 4°C. The supernatants were combined and an aliquot of 100 µl was transferred to a vial and dried in a vacuum (MiVac Duo concentrator, GeneVac Ltd, Ipswich, UK). Quality control samples were prepared by combining aliquots of samples from each plant line and each treatment. The vials were treated with nitrogen gas and stored at -80°C prior to derivatization and GC-MS analysis. The samples were redissolved in 40 µl of methoxyamine hydrochloride (MAHC, Sigma) (20 mg ml^-1^) in pyridine (VWR) and incubated for 90 min at 30°C at 150 rpm. The samples were then silylated with 80µl N-methyl-N-(trimethylsilyl) trifluoroacetamide with 1% trimethylchlorosilane (MSTFA with 1% TMCS, Thermo Scientific) for 90 min at 37°C at 150 rpm, and 100 µl of hexane (Sigma) containing alkane series (C10–C40, Supelco) was added to each sample.

### Metabolite Analysis by Gas Chromatography–Mass Spectrometry

The GC-MS system consisted of Agilent 7890A chromatograph system with Agilent 7000 Triple quadrupole mass spectrometer and GC PAL autosampler and injector (CTC Analytics). Sample (1 µl) was injected in splitless mode in a single tapered liner with glass wool (Topaz 4 mm ID Restek). Inlet temperature was set to 260°C. Helium flow in the column (Agilent HP-5MS Ultra Inert, length 30 m, 0.25 mm ID, 0.25 μm film thickness combined with Agilent Ultimate Plus deactivated fused silica, length 5 m, 0.25 mm ID) was 1.2 ml min^-1^ and purge flow was 46 ml min^-1^. MSD interface temperature was 180°C, MS source 230°C and quadrupole 150°C. The oven temperature program was as follows: 2 min at 50°C, followed by a 7°C min^-1^ ramp to 260°C, 15°C min^-1^ to 325°C, 4 min at 325°C and post-run at 50°C for 4.5 min. Mass spectra were collected with a scan range of 55-550 *m*/z. Deconvolution, component detection and quantification were conducted with Metabolite Detector (2.06 beta) (Hiller et al., 2009), and co-eluting components were confirmed with AMDIS (version 2.66, NIST). To confirm the identification of two detected metabolites, commercial standards of 3-hydroxy-3-methyl-glutarate (3-HMG), and 2-hydroxy-glutarate (2-HG) were analyzed. Metabolites were annotated based on standards or retention index and mass spectrum matched to databases and spectral libraries (Golm GMD database, Human Metabolome Database (HMDB), Fiehn GC/MS Metabolomics RTL library A.01.00, NIST, Wiley Registry MS-7^th^ edition). The relative contents of the metabolites were calculated by normalizing the peak areas by the peak area of the internal standard (ISTD, glycerol-d_8_) and the fresh weight (g) of the sample. The list of metabolites detected by GC-MS is in **Supplementary Table S1**. The redox status indicator ratios were calculated as in Kolbe et al. (2006) for isocitrate dehydrogenase (ICDH) or malate dehydrogenase (MDH). According to Kolbe et al. (2006) the NAD(P)-reduction state can be calculated with product/substrate ratio of NAD(P)-linked reactions like ICDH or MDH. The list of other NAD(P)H-producing dehydrogenases, with products and substrates were taken from Schertl and Braun (2014), and the ratios (product/substrate ratio) were calculated if both product and substrate were detected (**Supplementary Figure S1**).

### Histochemical Determination of Reactive Oxygen Species

The seedlings for histochemical detection were grown and exposed to MV identically to the seedlings for metabolite analysis as described in *Plant material*. Hydrogen peroxide and superoxide production were detected by staining plants with 3,3′-diaminobenzidine (DAB) as in Daudi et al. (2012) or with nitrotetrazolium blue (NBT) as in Jabs et al. (1996). After 16-h dark period (dark samples) or 12-h darkness overnight followed by 4.5 h of growth light, MQ water or MV solution was removed, and 1 ml of DAB or NBT solution was added to each well and infiltrated in a vacuum under dim light (<10 µmol m^-2^ s^-1^). Plants were then exposed to growth light (150 µmol m^-2^ s^-1^) for 30 min to stimulate ROS production, and/or kept in dark for 60 min, and then destained in 15 ml tubes.

### Statistical Analysis

Significant main effects of plant line, treatment and line × treatment interactions on individual metabolite levels and metabolite ratios were tested by two-way ANOVA with false discovery rate correction for multiple analysis, p-value < 0.05 considered significant (MassProfilerPro, Agilent). Significant effects on the metabolite levels were then visualized in Venn diagram. Principal component analysis (PCA) was performed to visualize general variation in the GC-MS data (MassProfilerPro). ANOVA-simultaneous component analysis was performed with 100 permutations to test the effects of plant line, treatment and their interaction (line × treatment) on the overall variation in the metabolite data [MetaboAnalyst, (Xia and Wishart, 2016)]. Orthogonal projections to latent structures discriminant analysis (OPLS-DA) was performed to study the differences of metabolite levels between the plant lines in control light conditions (Simca P+ version 15, Umetrics). In addition, OPLS-DA models were produced separately for *rcd1* and Col-0 comparing light to dark or to MV samples (see model diagnostics in **Supplementary Table S2**). S-plots were combined to produce shared and unique structures (SUS) plots to compare metabolite responses of *rcd1* and Col-0 to extended darkness or to MV treatment. PCA, OPLS-DA, and two-way ANOVA were performed without missing value imputation with log10-transformed data scaled by unit variance. Pathway analysis was performed to identify relevant metabolic pathways that differed between *rcd1* and Col-0 in control light conditions, after extended darkness and after MV treatment (MetaboAnalyst). Metabolic pathway enrichment analysis was based on 150 annotated metabolites in KEGG pathways. Data were log_10_-transformed, scaled by unit variance and missing values were imputed with k-nearest-neighbour (KNN) method. Pathway topology analysis was based on relative-betweenness centrality measurement and the significance of pathway enrichment analysis was tested with a global test algorithm. P-values derived from pathway enrichment analysis were natural base (e)-transformed.

## Results and Discussion

### Metabolite Profiles of *rcd1* and Col-0 Differ From Each Other and in Response to Methyl Viologen Treatment

**Figure 1:**
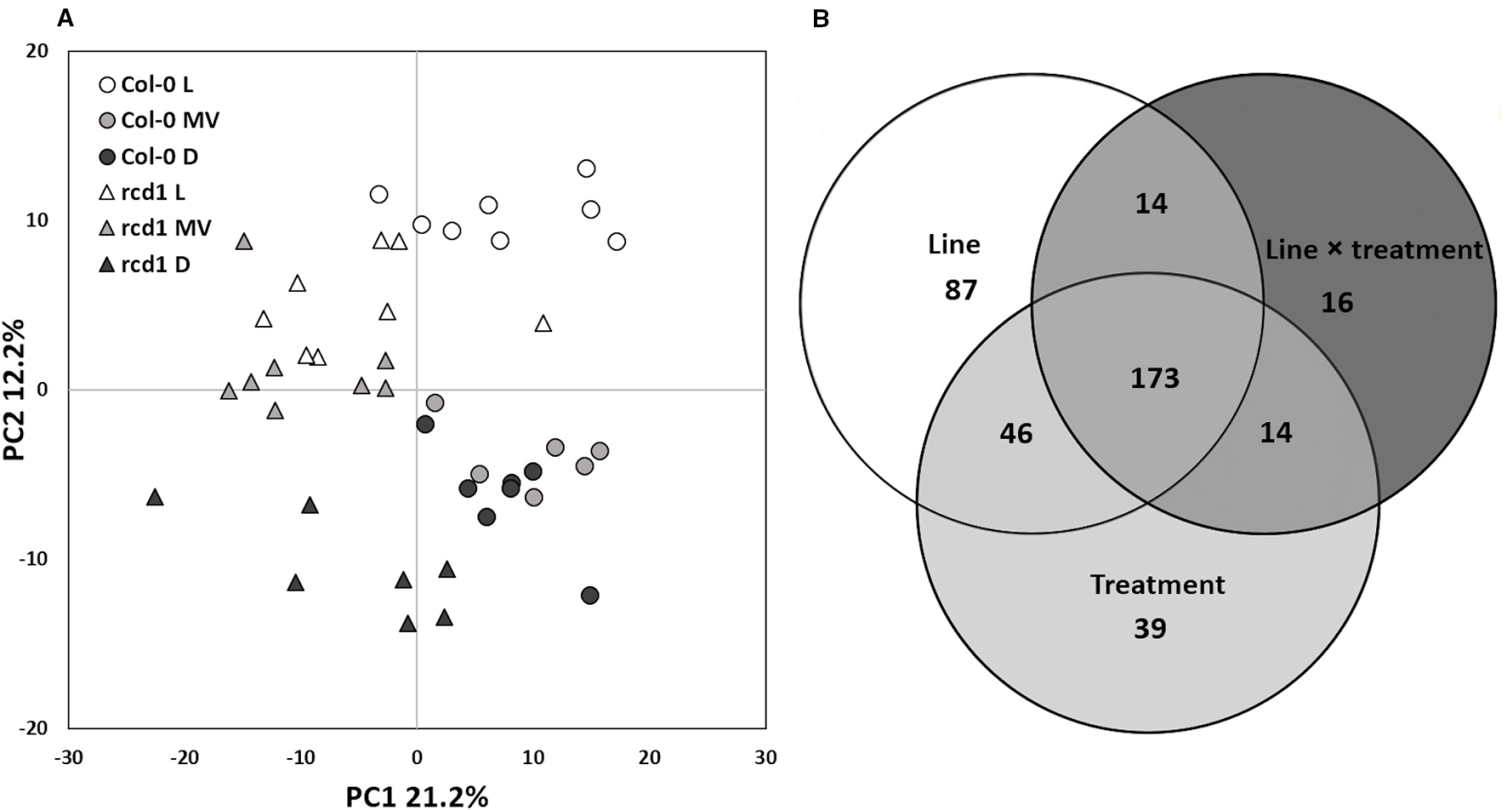
**(A)** Statistical analysis of GC-MS metabolite data of Col-0 and *rcd1* plants. Scores plot of principal component analysis of 489 metabolites. The first principal component separates the two plant lines and treatments are separated based on the second principal component, n = 6–8. Symbols and colors indicate different plant lines and conditions: circle and triangle indicate Col-0 and *rcd1*, and white, light gray and black indicate light/control (L), paraquat exposure (MV) and dark (D) conditions, respectively. **(B)** Venn diagram shows statistically significant effects of plant line (white), treatment (light gray) and line×treatment (dark gray) interaction on metabolite levels, two-way ANOVA, p < 0.05). The details of statistical results and the list of metabolites are in **Supplementary Tables S1** and **S3**.

**Figure 2:**
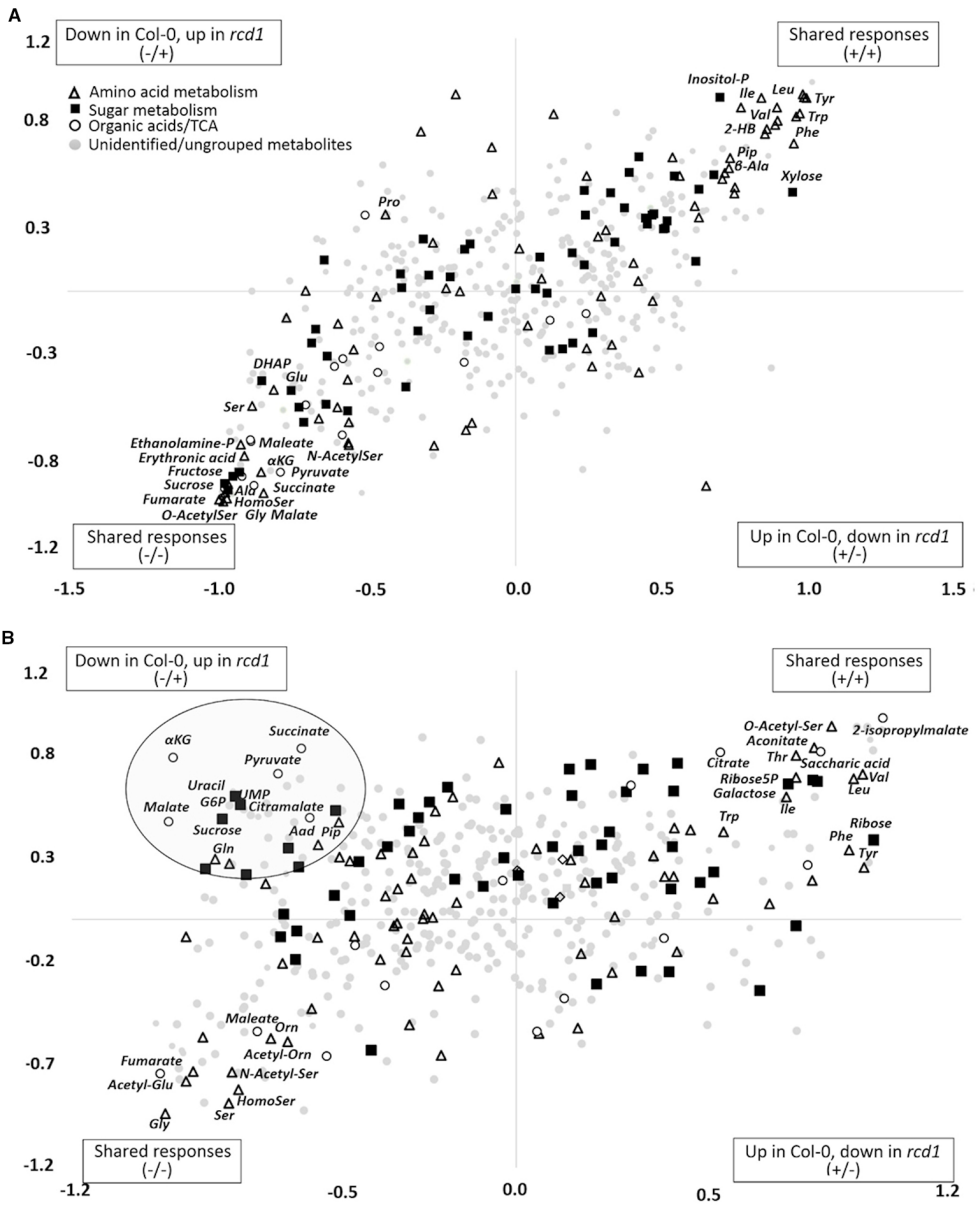
**(A)** Shared and unique structures (SUS)-plot of discriminant analysis (OPLS-DA) comparing metabolite responses of *rcd1* and Col-0 to extended darkness and to **(B)** MV treatment. Metabolites displaying similar responses in both plant lines are in upper right or lower left corner. Metabolites located along the axes display line-specific responses and metabolites located in the upper left or lower right corner display opposite responses in the plant lines. p(corr) is the OPLS-DA loading scaled as correlation coefficient. Dot shapes and colors indicate different metabolite groups. White triangle: amino acid metabolism, black square: sugar metabolism, white circle: organic acids/TCA cycle, light gray circle: unidentified/ungrouped metabolites (n = 6-8). The list of metabolite correlation coefficient values are in **Supplementary Table S6**.

Multivariate statistics were used to investigate the metabolite profiles of *rcd1* and wild type Col-0 plants in light (L), after MV exposure (MV) in light and after extended darkness (D) (**Figure 1**). The visual phenotypes of the two-week-old seedlings of *rcd1* mutant and Col-0 plants did not differ at the time of sampling (**Supplementary Figure S2**). However, based on their metabolite profiles, the plant lines were separated in PCA by the first principal component explaining 21.2% of the total variation (**Figure 1A**). Out of the 488 metabolites detected by GC-MS (plus starch), the levels of 320 metabolites were significantly different between the plant lines (**Figure 1B**, **Supplementary Table S3**). Samples from light conditions (L) and from extended darkness (D) were separated by the second principal component explaining 12.2% of the variation (**Figure 1A**). Since the responses to extended darkness were mainly similar in both plant lines (**Figures 1A** and **2A**), the large number of metabolites displaying significant line × treatment interaction (217 metabolites, **Figure 1B**) described primarily the different responses of *rcd1* and Col-0 to MV treatment (**Figure 2B**). In Col-0, MV samples were separated by the second principal component from the light samples and grouped close to the dark samples in PCA (**Figure 1A**). In marked contrast, MV samples of *rcd1* grouped close to the light samples (**Figure 1A**), which is in line with the MV-resistance of *rcd1*. The effects of line (p < 0.01), treatment (p < 0.01), and interaction term (p = 0.05) were significant in ANOVA-simultaneous component analysis (**Supplementary Figure S3**). Thus, the metabolite results were interpreted in the context of all three experimental conditions.

**Figure 3:**
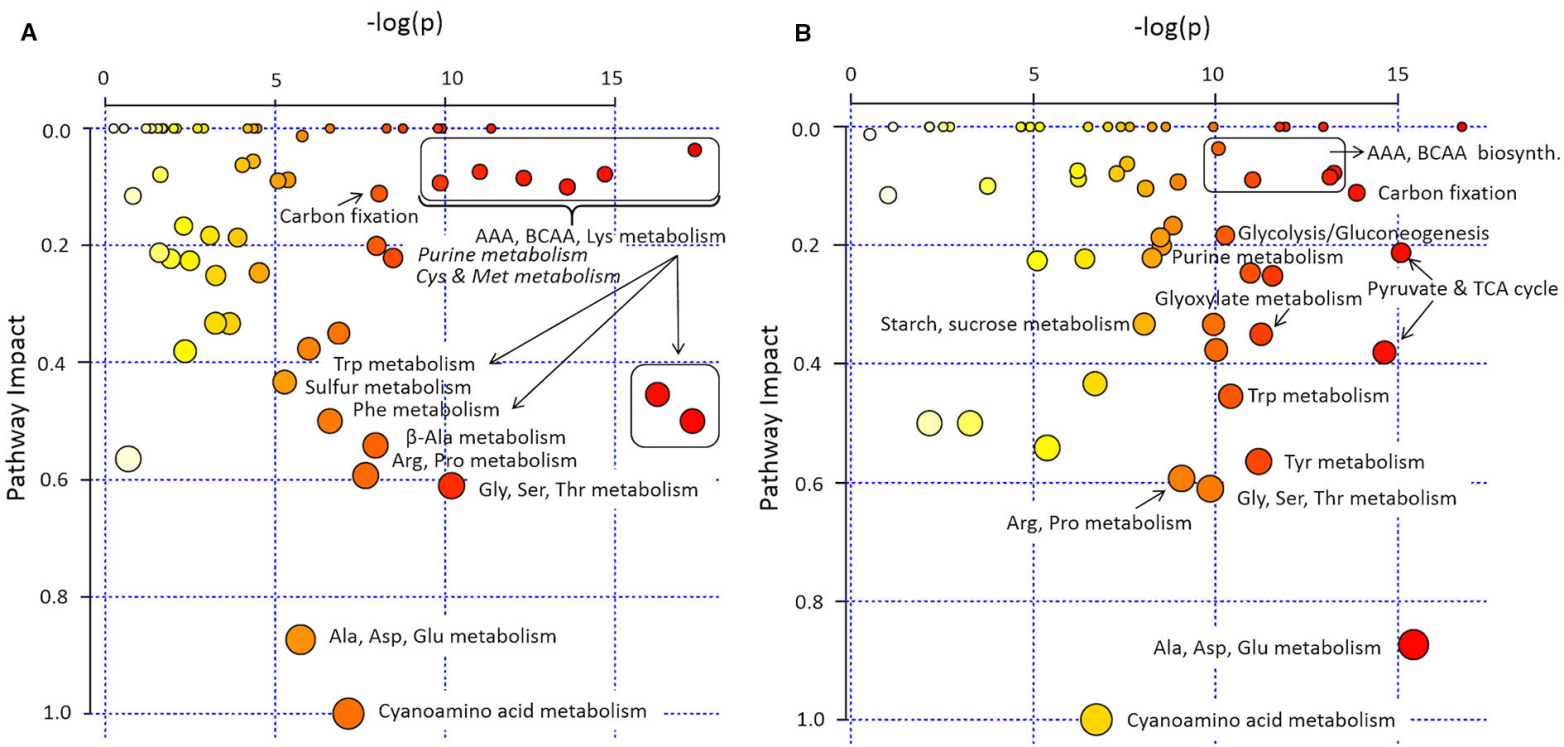
Pathway enrichment analysis of *rcd1* primary metabolite profile. **(A)** Light/control condition, **(B)** after 4h paraquat (MV) exposure in light (4 h light + 12 h in dark). Size of the node represents the intensity of the impact of plant line on the KEGG pathway based on the impact of each metabolite in a given pathway. Colour scale represents the significance of the pathway impact [-log(p); natural, e-base-log-transformation]. The list of pathways with their impact and -log(p) values are in **Supplementary Table S4**.

**Figure 4:**
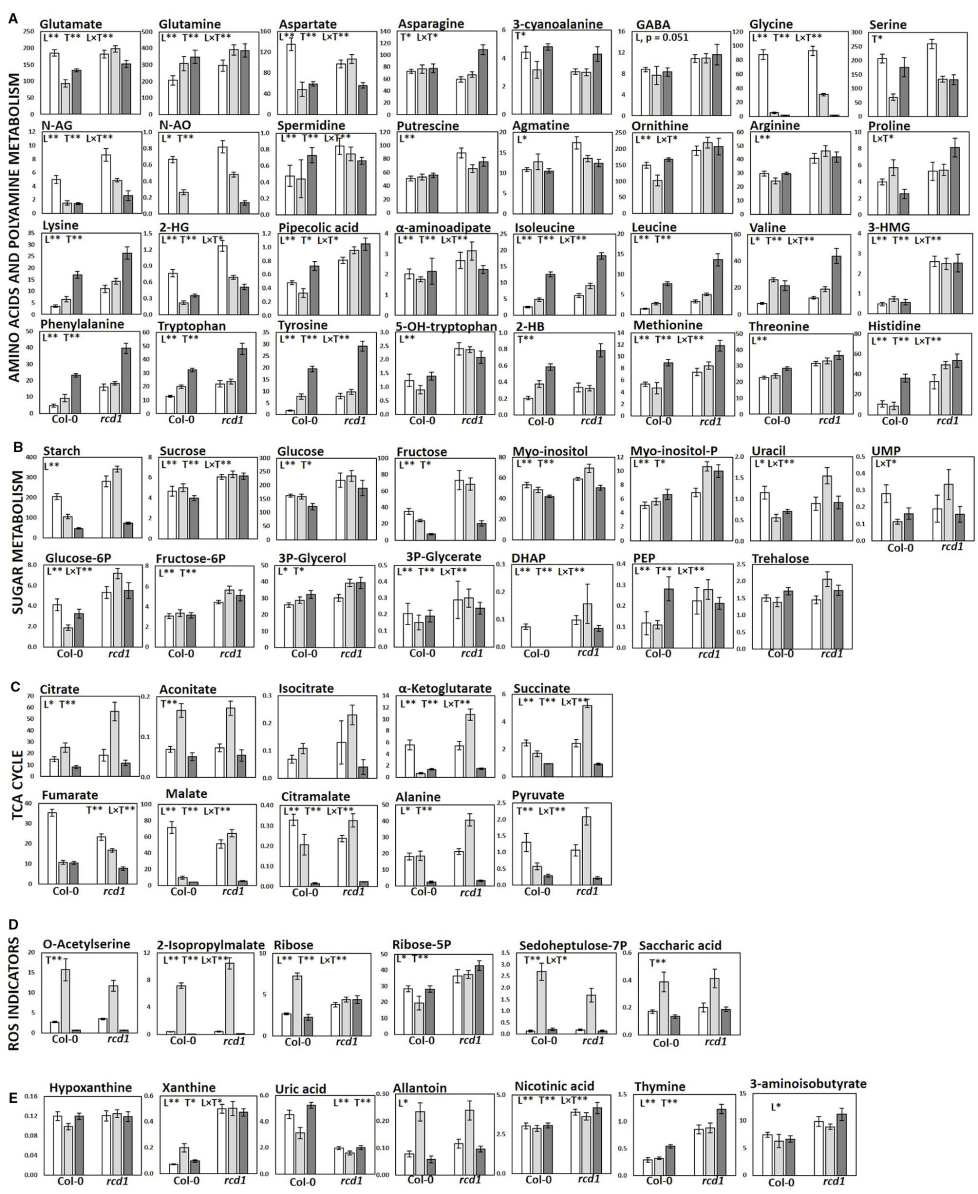
Metabolite levels in Col-0 and *rcd1* in control light conditions (white), after MV exposure (light gray) and after extended darkness (dark gray). **(A)** Amino acid and polyamine metabolism, **(B)** carbohydrate metabolism **(C)** TCA cycle, **(D)** ROS indicators and **(E)** nucleotide metabolism. Statistically significant main effects of plant line (L), treatment (T) and line × treatment interaction (L × T) on metabolite levels were tested with two-way ANOVA (p < 0.05*, p < 0.01**). Data are mean ± SE, n = 3–8, in arbitrary units (AU) mg^-1^ (FW).

**Figure 5:**
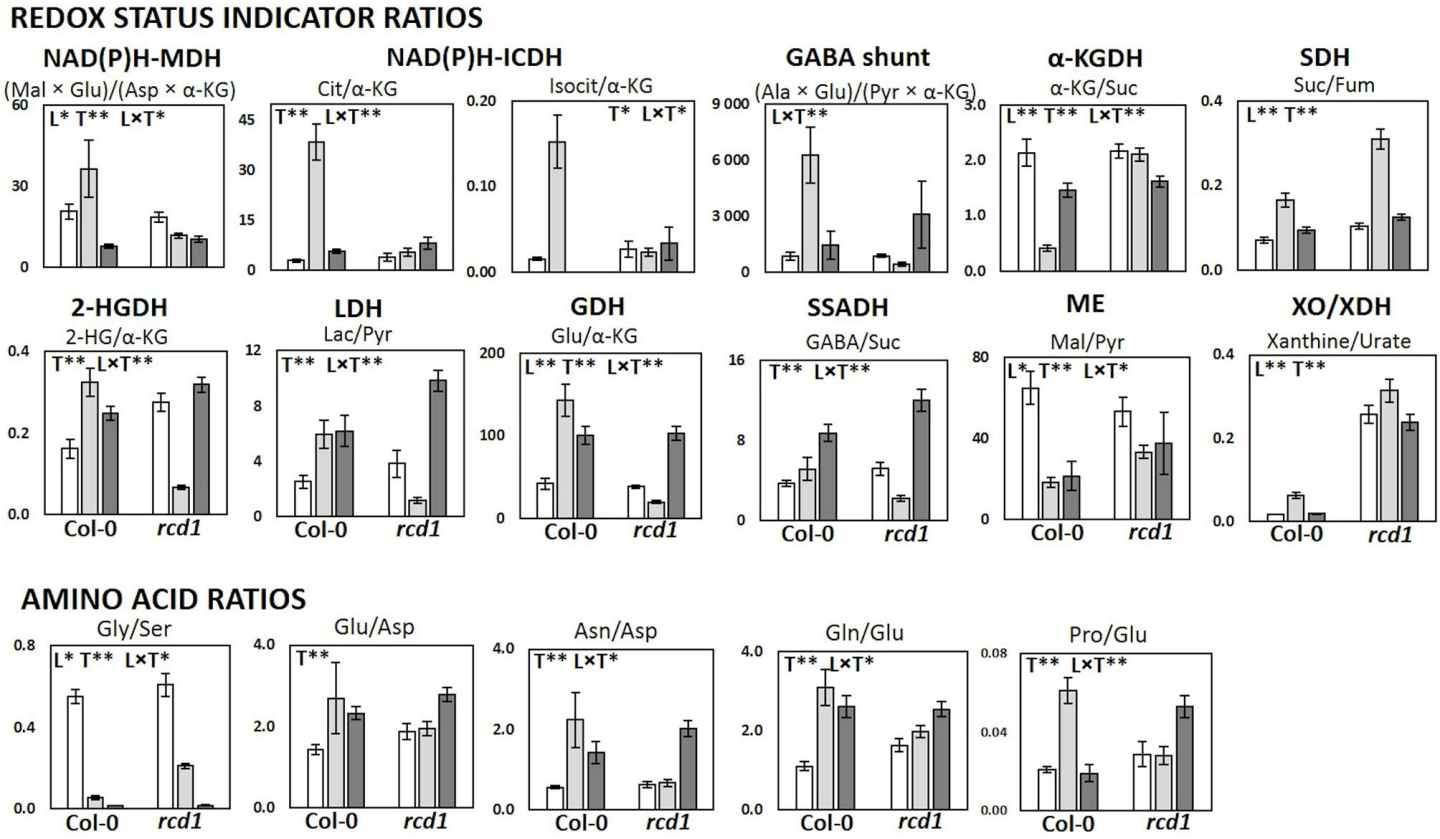
Metabolic and redox status indicator ratios in Col-0 and *rcd1* in control light conditions (white), after MV exposure (light gray) and after extended darkness (dark gray). Statistically significant effects of plant lines (L), treatment (T) and line × treatment interaction (L × T) on metabolite ratios were tested with two-way ANOVA (p < 0.05*, p < 0.01**). Data are mean ± SE, n = 3–8.

In pathway enrichment analysis, the metabolic pathways which were consistently enriched in *rcd1* irrespective of treatment, were connected to carbon fixation; aromatic amino acid and BCAA (+Lys) biosynthesis; purine, pyrimidine, nucleotide and ubiquinone metabolism; photorespiration and Thr metabolism; Arg and Pro metabolism; isoquinoline alkaloid as well as phenylpropanoid biosynthesis (**Figure 3**, **Supplementary Table S4**). In control conditions, several metabolite ratios describing photorespiration (Gly/Ser), nitrogen assimilation (Glu/Asp, Asn/Asp, Gln/Glu, Pro/Glu) and/or redox status [e.g. Cit/α-KG, 2-HG/α-KG, (Mal × Glu)/(Asp × α-KG)] were similar in both lines except xanthine/urate ratio, which was significantly higher in *rcd1* (**Figures 4** and **5**, **Supplementary Tables S1** and **S3**). The metabolite ratios had a similar response in both lines to extended darkness, except for Pro/Glu ratio, which showed no change in Col-0, while the ratio was 1.5-fold higher in *rcd1* in darkness than in light. The most significant differences between Col-0 and *rcd1* were detected in response to MV exposure (**Figures 4** and **5**). The two plant lines had an opposite response in amino acid and redox status indicator ratios, except Gly/Ser and xanthine/urate ratios (**Figure 5**). Both Gly/Ser and xanthine/urate ratios had similar response in both plant lines, but the response in *rcd1* was significantly weaker (Gly/Ser) and/or the ratio remained significantly higher (xanthine/urate).

### Altered Reactive Oxygen Species Production in *rcd1*

Seedlings were stained with DAB (3,3′-diaminobenzidine) and with NBT (nitro blue tetrazolium) to detect hydrogen peroxide (H_2_O_2_) and superoxide (O_2_^•-^) production, respectively (**Supplementary Figure S4**). NBT-staining revealed higher superoxide production in *rcd1* than in Col-0 in light conditions, yet no difference in superoxide production was observed in either of the plant lines in dark (**Supplementary Figure S4**). Induction of ROS production after 30 min exposure of light during staining, can already be seen in *rcd1* seedlings (**Supplementary Figures S4B, D**), indicating that superoxide production in *rcd1,* and the function of RCD1 are related to light-dependent processes. Increased superoxide production in *rcd1* has been published previously with older plants (Zhu et al., 2013; Shapiguzov et al., 2019), but not with young, 2-week old seedlings. However, no increase in H_2_O_2_ production was detected in *rcd1,* irrespective of age (**Supplementary Figure S4**) (Zhu et al., 2013; Shapiguzov et al., 2019). MV exposure caused a substantial increase of H_2_O_2_ in Col-0, but not in *rcd1* (**Supplementary Figure S4A**). By contrast, superoxide production slightly decreased in *rcd1* after MV exposure (**Supplementary Figures S4B, D**).

### Metabolic Features of *rcd1*

In control light conditions, pathway enrichment analysis showed that carbon fixation, amino acid metabolism and nucleotide metabolism were the most significantly changed in *rcd1* (**Figure 3A**). The levels of several glycolytic intermediates (sugars and sugar phosphates), amino acids [Aromatic; Phe, Tyr, Trp; BCAA's; Ile, Leu, Val; Lys; β-Ala; Met; His; Thr; Arg; GABA; N-acetyl-Glu (NAG); N-acetyl-Orn (NAO) and ornithine], and polyamines (spermidine, putrescine, agmatine) were higher in *rcd1* than in Col-0 (**Figure 4A**, **Supplementary Figure S5**, and **Tables S1**, **S3**, and **S5**). Starch content was slightly, but consistently, higher in *rcd1* than in Col-0 in all conditions (**Figure 4B**). In light, the glutamine/glutamate (Gln/Glu) ratio, an indicator of nitrogen assimilation, was higher in *rcd1* than in Col-0 (**Figure 5**), corresponding to the enhanced production of many nitrogen containing metabolites in *rcd1*. Low levels of asparagine (Asn) with its precursor, 3-cyano-alanine (**Figure 4A**) brought further evidence of altered N metabolism in *rcd1*. Mutant plants showed also higher levels of metabolites related to catabolism of Lys [pipecolate, 2-amino adipate, 2-HG, 2-hydroxy-butyrate (2-HB)] and Leu (3-HMG) (**Figure 4A**). The decreased metabolites in *rcd1* included citramalate and TCA cycle intermediates; fumarate and malate (**Figure 4C**), as well as metabolites of shikimate pathway: shikimate, *cis*- and *trans*-sinapates and -sinapoyl malates. Both the accumulation of AAA and BCAA metabolites and reduction of Asp, Asn, 3-cyano-Ala, fumarate, malate, citramalate and shikimate, sinapates and sinapoylmalates levels (**Figure 4**) have been associated with plant senescence in *Arabidopsis* (Watanabe et al., 2013). In addition, the free amino acid profile in *rcd1* resembled the changes in free amino acids that occur also during the induction of systemic acquired resistance in *Arabidopsis (* Návarová et al., 2012). Pathway enrichment analysis supported the results from individual metabolite data, and in addition to primary metabolism, also secondary metabolism such as phenylpropanoid biosynthesis was altered in *rcd1* (**Supplementary Table S4**). Nucleotide metabolism was significantly altered in *rcd1,* as the levels of most purines and pyrimidines analyzed, as well as metabolites related to nucleotide catabolism, were consistently higher in *rcd1* than in Col*-*0 regardless of the treatment (**Figure 4E**). The levels of xanthine, allantoin, inosine, nicotinic acid, 5-hydroxy-Trp, β-Ala, 3-aminoisobutyrate, thymine, guanine, adenosine and AMP were all higher in *rcd1* than in Col-0 (**Figure 4**, **Supplementary Tables S1** and **S3**). Only the levels of orotic and uric acid were lower in *rcd1* than in Col-0 (**Figure 4E**, **Supplementary Tables S1** and **S3**). Xanthine and uric acid levels are connected to redox regulated xanthine oxidase/dehydrogenase enzyme in cytosol (Ma et al., 2016), and the xanthine/urate ratio was significantly higher in *rcd1* than in Col-0 irrespective of the treatment (**Figures 4E** and **5**).

### Metabolite Responses to Extended Darkness

The *rcd1* mutant responded to extended darkness similarly with Col-0, with the levels of various metabolites remaining consistently higher as in light conditions (**Figures 2A** and **4**). Typical effects of extended darkness were seen in both plant lines in the levels of metabolites linked to glycolysis (sucrose, fructose, glucose, pyruvate, and sorbose), sucrose biosynthesis, photorespiration (glycine, serine, glycolate, and glycerate), TCA cycle (succinate, malate, fumarate, α-ketoglutarate, aconitate, and citrate) and protein catabolism (**Figures 2A** and **4**). Accumulation of AAAs and BCAAs and Lys, and decrease in Ala, Gly, Ser and Asp levels in both *rcd1* and Col-0 after prolonged night is a typical plant response to darkness (Araújo et al., 2010). Amino acids and their precursors accumulate due to inhibited protein synthesis, induced protein degradation, and because of delayed activation of mitochondrial degradation enzymes (BCAA's, AAA's and Lys) after illumination has ended (Araújo et al., 2010). The Gly/Ser ratio decreased in extended darkness, as photorespiration was inhibited (**Figure 5**). As Calvin cycle and CO_2_ assimilation are inhibited in darkness due to depletion of photosynthetic NADPH and ATP, the levels of precursors and intermediates from glycolytic pathway decreased, while the levels of hexose phosphates and glucose remained the same due to activation of light-regulated chloroplastic OPPP and starch degradation (**Figure 4B**). The levels of pyruvate and TCA cycle intermediates declined (**Figure 4C**), indicating not only the depletion in carbon supply to the TCA cycle, but also NADPH shortage from chloroplastic (light regulated) NADP-dependent malate-OAA-shuttle, which transfers reducing power to oxidative phosphorylation (COX). Also reduced DHAP levels can be seen in both lines as chloroplastic transport to cytosol *via* triose phosphate transporter (TPT) is inhibited in the dark (Dietz and Heber, 1984; Flügge, 1999), due to repressed conversion of GAP to DHAP as NADPH is needed as a substrate (**Figure 4B**). The most prominent differences between the dark responses in *rcd1* and Col-0 were seen in the levels of proline (Pro) and asparagine (Asn). Proline accumulated in *rcd1* plants, whereas in Col-0 the levels decreased in response to extended darkness (**Figures 2A** and **4A**). Asn accumulated in *rcd1*, but there were no significant changes in Col-0 (**Figures 2A** and **4A**). Enriched pathways after extended darkness in *rcd1* were related to AAAs, BCAAs (+ Lys), photorespiration and carbon fixation, as well as nucleotide, starch and sucrose metabolism, which were also enriched in the mutant in light conditions (**Figure 3A**, **Supplementary Table S4**). As the plant lines responded similarly to extended darkness, the function of RCD1 in the regulation of central carbon and nitrogen metabolism is mainly linked to light-dependent processes.

### The Possible Role of RCD1 in Central Carbon Metabolism

The consistently high levels of several glycolytic intermediates (**Figure 4B**, **Supplementary Figure S5**). indicated enhanced glycolytic activity in *rcd1* mutant that is supported by the previous data on transcriptomics (Brosché et al., 2014) and carbohydrate fluxes (Shapiguzov et al., 2019) The expression of three genes that encode glycolysis-related enzymes, 2,3-bisphosphoglycerate-independent phosphoglycerate mutase, cytosolic pyruvate kinase and fructose-bisphosphate aldolase (aldolase, FBA6) were up-regulated in *rcd1* (Brosché et al., 2014). Aldolase is shared between glycolysis and gluconeogenesis, while pyruvate kinase catalyzes the final step in glycolysis, dephosphorylating phosphoenolpyruvate (PEP) to pyruvate while generating ATP. As the glycolytic metabolite levels were high in *rcd1*, but pyruvate and TCA cycle intermediate levels did not correlate with the increase of glycolytic precursors in control conditions (L), we assume that the additional carbon flux is mainly redirected to cytosolic OPPP, and to starch and sucrose biosynthesis instead of mitochondria. This is consistent with the elevated levels of OPPP-derived metabolites (AAAs, His, sugars, and nucleic acids), starch and sucrose in *rcd1* mutant (**Figure 4** and **Supplementary Figure S5**). Additional evidence of changed carbohydrate metabolism comes from [U-^14^C] glucose feeding study that revealed enhanced carbon flux to starch and sucrose in *rcd1* (Shapiguzov et al., 2019). In addition to increased glycolytic activity, the elevated levels of OPPP-derived metabolites and sugars in *rcd1* under light conditions suggest increased OPPP activity that can lead to more reductive conditions in the cytosol through the production of NAD(P)H. Cytosolic and chloroplastic OPPPs contribute to redox homeostasis and ROS scavenging through NADPH production, but are regulated in a different manner (Hauschild and von Schaewen, 2003; Couée et al., 2006; Bolouri-Moghaddam et al., 2010; Esposito, 2016). The cytosolic OPPP activity stays rather constant in light or dark conditions, but can be induced by various stresses, continuous light, or in darkness, if metabolizable sugars are available (Hauschild and von Schaewen, 2003; Esposito, 2016). By contrast, the activity of the chloroplastic OPPP is normally inhibited in light, but in darkness the inhibition is removed by redox regulation (Esposito, 2016), and in response to MV treatment, the activity of plastidic OPPP was elevated, but the activity of cytosolic OPPP did not change (Hauschild and von Schaewen, 2003).

Directing carbon flow between glycolysis and cytosolic OPPP may involve also redox control through the key regulators of glycolysis and energy metabolism. In the plant cytosol, the NADPH for carbon reduction is mainly provided by three enzymes, NADP-dependent isocitrate dehydrogenase (IDH), glucose-6-phosphate dehydrogenase (G6PDH, OPPP), and NADP-glyceraldehyde-3-phosphate dehydrogenase (GAPN) (Noctor, 2006). The NAD-dependent GAPDH (GAPDH), is highly sensitive to ROS (Schneider et al., 2018) and the H_2_O_2_-induced oxidative stress has been reported to increase mitochondrial association of cytosolic AtGAPDHs as well as their transport to the nucleus (Sweetlove et al., 2002; Zaffagnini et al., 2013). As there is another non-phosphorylating, NADP-dependent glyceraldehyde dehydrogenase (GAPN), which is less sensitive to ROS, the continuous, but diminished carbon flux to TCA cycle during oxidative stress can continue. Increased activity of cytosolic, NADPH-producing GAPN has been observed in maize and in wheat after MV exposure (Bustos et al., 2008). Also cytosolic thioredoxins maintain GAPDHs in active, reduced state and thioredoxin expression (trx-h5) is induced by ROS generation, such as in Arabidopsis Col-0 plants exposed to MV (Scarpeci and Valle, 2008). However, cytosolic thioredoxin-h5 (trx-h5) expression was down-regulated in *rcd1* (Brosché et al., 2014) that may impair the regulation of cytosolic redox status in addition to increased ROS production and glycolytic metabolites (and redirected carbon flow to OPPP). Also slightly decreased/similar levels of TCA cycle intermediates in *rcd1* could be explained by these “moonlighting” properties of glycolytic enzymes.

### RCD1 Is Involved in Nitrogen Metabolism

The most consistent and significant differences in pathway enrichment analysis in *rcd1* when compared to Col-0 were connected to nitrogen metabolism, mainly to metabolism of AAAs, BCAAs and Lys, Gly, Ser and Thr as well as nucleoside (purine, pyrimidine) metabolism in all three treatments (L, D, MV) (**Figure 3**, **Supplementary Table S4**). α-Ketoglutarate (α-KG) is not only an intermediate of the TCA cycle, but also a key carbon backbone donor (precursor of Glu) for several amino acids integrating carbon and nitrogen metabolism. On the other hand, Glu functions also as an amino-group donor for amino acids. While sugar and sugar-P levels (C metabolism) were consistently increased in *rcd1* (**Figure 4B**), there were no distinct accumulation of α-KG, adjacent TCA cycle intermediates, or Glu and Gln in control conditions. However, majority of amino acid levels were increased in *rcd1*, suggesting that the excess C (and N) are directed to amino acid and nucleoside biosynthesis, instead of TCA cycle. *Rcd1* mutant displayed low levels of only three amino acids; cyano-alanine, Asp and Asn, while downstream metabolites Lys, Met, Ile, and Thr levels were high (**Supplementary Figure S5**). This suggests inhibited conversion of Asp to Asn, and enhanced carbon flow to Lys and other downstream metabolites. On the other hand, in extended darkness, *rcd1* displayed high Asn levels. Altered Asp and Asn levels in *rcd1* can be explained by the high expression of cytosolic aspartate amino transferase (AAT2, Brosché et al., 2014) that has been shown to coordinate the biosynthesis of Asp in light and its conversion to Asn in the dark (Schultz et al., 1998). AAT2 catalyzes also the reversible transamination reaction of Glu and oxaloacetate (OAA) to Asp and α-ketoglutarate, connecting nitrogen metabolism to pivotal carbon flux (TCA cycle), and to shuttling of reducing equivalents between mitochondria and cytosol (malate-Asp shuttle) (Wilkie and Warren, 1998). In a similar manner, the excess glycolytic carbon flux (and N assimilation *via* Gln) could be directed to amino acid biosynthesis *via* C3 metabolites (PEP, pyruvate, 3-PGA) instead of TCA cycle.

The significant accumulation of AAAs is a typical plant response to dark-induced senescence, to systemic acquired resistance, defense priming and to abiotic stress (Araújo et al., 2010; Návarová et al., 2012; Watanabe et al., 2013) due to activated biosynthesis (chloroplast) as well as inhibited catabolism (mitochondria). Shikimate route enzymes can also be activated by the loss of RCD1/STO-inhibition of COP1/HY5 pathway (Jiang et al., 2009). The accumulation of BCAAs followed the same pattern as AAA's in *rcd1*. The biosynthesis of both BCAAs and AAAs requires carbon backbone precursors that are derived from OPPP and glycolysis, NADPH as a substrate and Glu as an amino donor. Thus, surplus of chloroplastic NADPH and excess of cytosolic OPPP derived precursors likely contribute to the constantly high levels of AAAs and BCAAs in *rcd1*.

### RCD1 Plays a Role in Regulating Nucleotide Metabolism and Cellular Redox Homeostasis

In addition to the elevation of glycolytic intermediate and OPPP-derived metabolite levels, altered xanthine/urate ratio supports the hypothesis that cytosolic environment is more reduced in *rcd1* than in Col-0 (**Figure 5**). Xanthine oxidase/dehydrogenase (XO/XDH) is an enigmatic enzyme, which has both oxidase (XO) and dehydrogenase (XDH) activities (Vorbach et al., 2003). In plants, the opposing roles have been reported to be regulated by spatially distinct substrate availability (Ma et al., 2016). In presence of NAD^+^, XDH is activated and enzyme acts as a dehydrogenase taking part in purine catabolism by converting xanthine to urate in the cytosol. Urate is transported to peroxisomes for degradation through allantoin, which is further catabolized in endoplastic reticulum to glyoxylate, ammonia and carbon dioxide. In the presence of NADH, XO is activated and enzyme acts as an oxidase, producing ROS (O_2_^•^¯) from O_2_ but with no or inhibited conversion of xanthine to urate (Ma et al., 2016). The levels of xanthine, urate, and glycolytic metabolites suggest that cytosolic NAD(P)H levels could be higher in *rcd1* than in Col-0, and that in *rcd1* the XO/XDH acts predominantly as an oxidase. The ability of XO to be rapidly converted to XDH under various forms of stress and damage makes it an ideal component for fast innate immune and oxidative stress responses. *Atxdh1* mutant displays xanthine accumulation, premature senescence and extensive cell death (Brychkova et al., 2008) mirroring the phenotypes of *rcd1.*

Elevated superoxide production in *rcd1* was detected only under light indicating that ROS have to derive from light-related processes (**Supplementary Figure S4**). Shapiguzov et al. (2019) proposed that RCD1 is involved as a co-regulator in acclimation and adjustment-related processes that affect chloroplastic redox status *via* mitochondrial processes. Highly reduced state of 2-cysteine peroxiredoxin (2-CysPrx) and activated NADPH-dependent malate dehydrogenase (NADPH-MDH) in *rcd1* seedlings suggested reduced availability of electron acceptors and activation of transfer of reductive power out of chloroplasts and altered ferredoxin/thioredoxin (Fd/TRX) regulation. Several chloroplastic OPPP and Calvin cycle enzymes are also redox regulated by Fd/TRX system (Entus et al., 2002). Interestingly, MV exposure reduced superoxide production in *rcd1,* but with no adjoining accumulation of H_2_O_2_ from superoxide scavenging as seen in Col-0. This may suggest that over-reduced environment in *rcd1* could account for the observed higher rate of O_2_^•^¯ generation, and the introduction of highly effective redox cycler (paraquat, MV), which limits the pool of intracellular reductant (NAD(P)H) levels (Cochemé and Murphy, 2009), limits also following O_2_^•^¯ production in *rcd1*.

In contrast to MV exposure, the levels of metabolite markers of chloroplastic or mitochondrial oxidative stress were not elevated in *rcd1* in control light conditions. This implies that higher cytosolic NAD(P)H levels may enhance extracellular ROS production in *rcd1 via* NADPH-oxidase family enzymes in the plasma membrane (Respiratory Burst Oxidase Homologs, RBOH) or in other cellular compartments (vacuole, endoplasmic reticulum (ER) or nuclei), and contribute to the elevated superoxide levels (Torres and Dangl, 2005). Reductive environment in the cytosol and the concurrent production of extracellular ROS *via* NADPH oxidases could also explain the hypersensitivity of *rcd1* to ozone and apoplastic superoxide (Overmyer et al., 2000).

The metabolite features of *rcd1* mutant suggested changed anabolic and catabolic processes connected to purine and pyrimidine metabolism. The levels of UMP and uridine were high in *rcd1* after MV treatment indicating activation of pyrimidine (uridine) salvage pathway. Uridine nucleotides can be formed by energy-consuming *de novo* synthesis or by the energy-saving recycling of nucleobases resulting from nucleotide catabolism (Mainguet et al., 2009). Activation of uridine salvage pathway in *rcd1* is supported by the high transcript levels of UKL2 in *rcd1* (Brosché et al., 2014). Uridine kinase (UKL2) is involved not only in uridine salvage pathway, but also in starch, sucrose (via UDP-glucose) and lignin biosynthesis (Chen and Thelen, 2011).

In *Arabidopsis*, NAD is synthetized from Asp, and besides redox reactions NAD can act as a substrate for generation of ADP-ribose, poly(ADP-ribos)ylation and deacetylation of proteins (Hashida et al., 2009). During those processes, NAD is degraded and must be recycled in NAD salvage pathway, which is known to play a key role in e.g. plant abiotic stress tolerance (Li et al., 2015). Several metabolites connected to NAD biosynthesis and salvage pathway (e.g. nicotinic acid, AMP, Rib-5-P) were higher in *rcd1*, except precursor Asp, which was significantly decreased in *rcd1*, which suggests that the pathway is influenced by RCD1. Two UDP-glucosyl transferase family protein transcripts (UGT74F2, UGT76B1/SGAT1) are down-regulated in *rcd1* (Brosché et al., 2014). The enzymes transfer glucose from UDP-glucose to nicotinic acid in a NAD salvage pathway and accordingly *ugt74f2* mutant accumulates free nicotinic acid (Li et al., 2015). Further evidence of altered nucleotide metabolism in *rcd1* stems from transcriptomic study that showed altered expression of genes that are connected to NAD metabolism (NUDX6; NUDX10) and PARP activity (PARP2) (Brosché et al., 2014). NUDIX hydrolases have also been associated with the prevention of excessive accumulation of NADH and inhibition of oxidative stress. AtNUDX enzymes cleave either NADH or ADP-ribose (Ogawa et al., 2005), and both NUDX6 (down-regulated in *rcd1*) and NUDX10 (up-regulated in *rcd1*) show activity as ADP-ribose pyrophosphatase. Only NUDX6 shows substantial activity to NADH and the intracellular NADH levels are high in the *nudx6* knock-out mutant (Ogawa et al., 2016). In addition, NUDX6 has a link to the regulation of cytosolic redox status as it induces the expression of TRX-h5. Accordingly, the transcript levels of both NUDX6 and TRX-h5 were low and the levels of PARP2 transcripts were elevated in *rcd1* (Brosché et al., 2014). Taken together, these findings suggest that RCD1 is involved in nucleotide metabolism, NAD metabolism and cellular NADH levels.

### Accumulation of Senescence Markers in *rcd1*

Senescence-associated metabolites (**Figure 4**, **Supplementary Figure S5**) accumulated in *rcd1* already at an early developmental stage while senescence-related gene expression was mainly down-regulated in *rcd1* (Brosché et al., 2014). This indicates that the early senescence observed in *rcd1* mutants is an atypical phenomenon. The *rcd1* mutant accumulated hydroxy acids (e.g. 3-HMG, 2-HG, 2-HB) that arise from mitochondrial degradation of Lys and BCAAs. HMG-CoA (3-hydroxy-3-methyl-glutaryl-CoA) is an intermediate in both mitochondrial Leu catabolism and cytosolic mevalonate (MEV) pathway, whereas 3-HMG is a degradation product of HMG-CoA with unknown function and regulation in plants. The altered metabolism of BCAAs and AAAs can be seen also in significantly elevated levels of 3-HMG (6-fold) in *rcd1*. In plants, the accumulation (10-fold) of 3-HMG have been detected in senescing leaves (Watanabe et al., 2013), but its function has remained unclear. In mammals, a defect in HMG lyase in mitochondria has been reported to cause breakdown of HMG-CoA to toxic 3-HMG, instead of conversion of HMG-CoA to acetoacetate and acetyl-CoA (Faull et al., 1976; Puisac et al., 2010). The defect in HMG lyase and the accumulation of 3-HMG enhance ROS formation, lipid peroxidation and xanthine oxidase activity and inhibit mitochondrial respiratory chain (COX) by inactivation of Cyt c (Struecker da Rosa et al., 2016). 2-Hydroxy acids play an integral role in plant metabolism and are involved in several fundamental pathways, including photorespiration, TCA cycle, glyoxylate cycle, methylglyoxal pathway and lysine catabolism. 2-Hydroxyglutaric acid (2-HG) is an end product of mitochondrial Lys catabolism (Araújo et al., 2010; Engqvist et al., 2011; Engqvist et al., 2014) and like 3-HMG, it is also reported to be a metabolic marker for mitochondrial dysfunction, to induce oxidative stress and to perturb energy homeostasis in mammals (Gelman et al., 2018; Ye et al., 2018). 2-HG belongs to a group of known senescence markers in plants together with AAAs, BCAAs, pipecolic acid and α-amino adipic acid (Engqvist et al., 2011; Watanabe et al., 2016), all of which showed elevated levels in *rcd1*. 2-HG accumulation causes dose-dependent inhibition of cytochrome c oxidase activity (complex IV), yet in plants, no specific phenotype or toxic effects have been related to high levels of 2-HG (Hüdig et al., 2015). The accumulation of senescence markers and impaired catabolism of amino acids in mitochondria could be related to the function of RCD1 in coordinating genes in mitochondrial dysfunction stimulon through ANAC013 and ANAC017 interaction, or possibly caused indirectly by other factors, such as increased ROS production or redox imbalance.

### Metabolite Responses to Methyl Viologen and Possible Resistance Mechanisms

#### Differing Metabolite Responses to the Methyl Viologen—Col-0 vs *rcd1*

The metabolite responses to the MV exposure differed greatly between *rcd1* and Col-0 plants (**Figures 2** and **5**). In *rcd1*, pathways related to TCA cycle and pyruvate metabolism, carbon fixation, glycolysis, starch and sucrose, inositol phosphate, amino acid and nucleotide metabolism were most significantly enriched after MV exposure (**Figure 3B**). Accordingly, the levels of many sugars and sugar phosphates (sucrose, glucose-6-P, fructose-6-P, PEP, DHAP, inositol, inositol-P, and trehalose) showed different responses to MV treatment in *rcd1* and Col-0 (**Figures 2B** and **4B**). However, the most prominent differences between Col-0 and *rcd1*, when comparing the control conditions to MV-exposure in the pathway enrichment analysis, were the pyruvate and TCA cycle metabolism, and glycolysis/gluconeogenesis (**Figures 3A, B**).

In Col-0 after MV exposure, citrate and aconitate levels were higher and the levels of metabolites downstream in TCA cycle were lower than in control conditions (**Figures 2B** and **4C**). Although the principal site of action of MV in plants is in chloroplasts (PSI), clear indications of increased ROS production was observed in both chloroplastic and mitochondrial oxidative stress marker levels (**Figures 4C, D**). The accumulation of citrate and aconitate, as well as the decline in the levels of downstream metabolites of TCA cycle indicated inhibition of mitochondrial aconitase and respiration in Col-0, as aconitase is known to be highly sensitive to ROS, particularly to H_2_O_2_ and superoxide (Verniquet et al., 1991). By contrast, in *rcd1*, the levels of all detected TCA cycle intermediates, except fumarate, as well as Ala, pyruvate and citramalate increased or remained high after MV exposure (**Figures 2B** and **4B, C**). Citramalate is not an actual intermediate of TCA cycle, but after it was detected in *Arabidopsis* by Fiehn et al. (2000), it has been suggested that there might be a similar kind of TCA cycle bypass also in *Arabidopsis* as there is in bacteria (Grant and Smith, 2000). The levels and responses of citramalate correlated with malate, succinate, pyruvate, α-KG levels and even Ala (originates from pyruvate) in both plant lines, suggesting a tight connection between citramalate and TCA cycle. In addition, the levels of Asp and Glu, which are linked to mitochondrial malate-aspartate shuttle, followed the same response patterns as the TCA cycle intermediates (**Figures 4A, C**).

The metabolite ratios, which have been previously used as redox status indicators in Kolbe et al. (2006) or describing the function of NAD(P)H producing mitochondrial enzymes (Schertl and Braun, 2014) differed between Col-0 and *rcd1* during MV exposure. The metabolite ratios indicating activities of malate dehydrogenase (NAD(P)H-MDH), isocitrate dehydrogenase (NAD(P)H-ICDH), glutamate dehydrogenase (GDH), 2-hydroxyglutarate dehydrogenase (2-HGDH), and lactate dehydrogenase (LDH) as well as amino acid Pro/Glu ratio were elevated in Col-0 during MV-induced oxidative stress, while the ratios declined or had no significant change in *rcd1* (**Figure 5**, **Supplementary Tables S1** and **S3**). The product(s)/substrate(s) ratios related to GABA shunt and Ala aminotransferase (ALAT) had also a similar response to MV as NAD(P)-MDH, NAD(P)H-ICDH, GDH, D-2HGDH, and LDH. The metabolite ratio for α-KG dehydrogenase (α-KGDH) behaved in an opposite manner, decreasing in Col-0 and increasing in *rcd1* during the MV exposure (**Figure 5**, **Supplementary Tables S1** and **S3**). The differing responses to MV in both individual TCA cycle metabolite levels as well as the redox status ratios indicate sustained respiration flux and increased glycolytic activity to produce pyruvate to feed the TCA cycle in *rcd1* during MV exposure.

In addition to altered TCA cycle metabolite levels, the decline of sucrose, fructose, glucose-6P and mannose-6P levels is in line with reduced photosynthetic activity, glycolysis, and sucrose biosynthesis in MV-sensitive Col-0 after MV exposure (**Figure 4B**) as well as with MV- (Scarpeci and Valle, 2008) and menadione-induced oxidative stress responses (Baxter et al., 2007; Lehmann et al., 2009). In addition, the majority of pyrimidine nucleotide, nucleobase and their catabolite levels were reduced during MV exposure in Col-0 (**Figure 4E**). However, the levels of sucrose, glucose-6P, UMP and uracil levels increased in *rcd1* during MV exposure mirroring the responses of TCA cycle metabolites, while the majority of detected sugar levels in *rcd1* remained high (**Figure 4B**). AMP, ribose and adenosine levels were elevated in Col-0 after MV exposure, which could be due to ROS-induced increased PARG (Poly-(ADP-Ribose) Glycohydrolase) or NUDIX (nucleoside diphosphate-linked moiety X hydrolase) activity, producing AMP and ribose from polyADP ribose (Formentini et al., 2009). The high content of soluble carbohydrates and starch in *rcd1* indicated that carbon fixation was not hindered in the mutant plants after MV exposure. Thus, we can assume that there is no severe NADPH depletion in *rcd1* chloroplasts due to the MV-redox cycle, and the Calvin cycle continues to assimilate CO_2_ generating sugars. Unlike in Col-0, chloroplastic OPPP is not activated, however cytosolic OPPP continues to function, as it is not as sensitive to redox regulation as its corresponding plastidic pathway (Scarpeci and Valle, 2008; Esposito, 2016). The activity of cytosolic OPPP is presumably increased in *rcd1* due to enhanced glycolytic activity and elevated hexose levels (Hauschild and von Schaewen, 2003). Both glycolysis and OPPP generate reductive power (NAD(P)H) that can feed the antioxidant system and ROS processing under MV-induced ROS generation in the cytosol and other cellular locations as a result of efficient transfer of reducing equivalents between organelles.

The Gly/Ser ratio, a classic indicator of photorespiratory activity (Wingler et al., 1999; Wingler et al., 2000), decreased in both plant lines after MV exposure: a 10-fold decrease in MV-treated Col-0 plants, but only 3-fold decrease in *rcd1* plants (**Figure 5**). In Col-0, the strong decline in Gly/Ser ratio during MV exposure relates to the decrease in photosynthetic activity, reduced NADPH/NADP ratio and mitochondrial damage (glycine decarboxylase inhibition) (Taylor et al., 2002; Noguchi and Yoshida, 2008), whereas in *rcd1*, the levels of glycolytic intermediates (and sucrose) stayed elevated and photosynthetic activity was reported to continue during MV exposure (Shapiguzov et al., 2019). As well as the enhanced glycolysis and TCA cycle metabolite results in *rcd1* during MV exposure, the decline in Gly/Ser ratio could be related to the increase in mitochondrial NADH/NAD^+^ ratio that inhibits glycine decarboxylase complex (Noguchi and Yoshida, 2008; Watanabe et al., 2016).

#### Increase in Common Stress Markers Indicate Oxidative Stress in Both Plant Lines

Apart from significantly different metabolite responses to MV between *rcd1* and Col-0, the two plant lines exhibited also common responses to MV (**Figure 2B**, **Supplementary Table S6**). Common responses to MV included a decrease in the levels of ornithine precursors, N-acetyl-glutamate (NAG) and N-acetyl-ornithine (NAO) (**Figure 4A**) and an accumulation of oxidative stress markers 2-isopropylmalate and O-acetyl serine (OAS), and of OPPP intermediates ribose-5P and sedoheptulose-7P, and saccharic acid, an oxidized form of glucose (**Figures 2B** and **4D**). Similarly to aconitase, its chloroplastic homolog, isopropylmalate isomerase (IPMI), is highly sensitive to ROS (Ellerström et al., 1992). IPMI is involved in Leu biosynthesis and it catalyzes the isomerization of 2-isopropylmalate to 3-isopropylmalate. Inhibition of IPMI and the subsequent accumulation of 2-isopropylmalate were evident in both plant lines after MV exposure (**Figure 4D**). This indicated that MV reached its primary site of action and triggered the overproduction of ROS in the chloroplasts in both plant lines. Another indicator of oxidative stress was the accumulation of a known oxidative stress marker, O-acetyl serine (OAS), which is related to sulfur and cysteine biosynthesis distributed across cytosol, mitochondria and chloroplast (Saito, 2004; Kawashima et al., 2005; Lehmann et al., 2009) (**Figure 4D**). The cyclic ornithine pathway in planta is localized in the chloroplast and consumes both ATP and NADPH (Chen et al., 2006). As the N-acetylglutamate kinase (NAGK) uses ATP and N-acetylglutamyl-5-P reductase (NAGPR) uses NADPH as a substrate, depletion of both ATP and NADPH during extended darkness and MV-induced oxidative stress in chloroplasts inhibits the cyclic pathway, and the levels of ornithine precursors, N-acetylglutamate (NAG) and N-acetyl-ornithine (NAO) decrease (Slocum, 2005), which can be seen in both plant lines (**Figure 4A**). The accumulation of ribose-5P (R5P), sedoheptulose-7P (Sedo-7P) and metabolites derived from E4P and R5P, suggest OPPP activation (Baxter et al., 2007; Lehmann et al., 2009) in both lines. Also the clear accumulation of saccharic acid (**Figure 4D**), an oxidized form of glucose, was detected in both lines. These metabolite responses are characteristic under enhanced generation of ROS (Baxter et al., 2007; Lehmann et al., 2009; Noctor et al., 2015), which suggests that the MV-resistance in *rcd1* is a result of altered cellular metabolism and not related to diminished delivery of MV into the cells or chloroplasts.

#### Role of TCA Cycle and Mitochondrial Processes to Methyl Viologen Tolerance

Previous studies have shown that the flux through mETC (mitochondrial Electron Transport Chain) is altered in *rcd1*, as respiration through alternative oxidases (AOX) is strongly enhanced (Brosché et al., 2014; Shapiguzov et al., 2019). Moreover, potassium cyanide (KCN), an inhibitor of complex IV, decreased oxygen consumption in Col-0 significantly, but did not affect oxygen consumption in *rcd1* (Shapiguzov et al., 2019). Respiration through AOX is less efficient in ATP production due to inhibited proton pumping in complexes III and IV (COX) causing decreased proton gradient across the membranes. Thus, enhanced respiration flux through AOXs in *rcd1* could lead to the induction of alternative ATP producing pathways such as glycolysis and energy-saving salvage pathways, which can be seen in the metabolomic profile of *rcd1*. Apart from transcriptional control of their expression, AOX isoforms in *Arabidopsis* are redox regulated and their activity and transcription is induced by high pyruvate and/or isoform-specific keto acids (Selinski et al., 2018), of which pyruvate and α-KG were elevated in *rcd1* in MV treatment. Also, high non-phosphorylating respiration flux through AOX is a consequence of increased NADH levels and the elevated expression of alternative NAD(P)H dehydrogenases on the mitochondrial inner membrane (Clifton et al., 2006; Elhafez et al., 2006). Elevated expression of external alternative NAD(P)H-ubiquinone oxidoreductase B3 (NDB3) in *rcd1* (Brosché et al., 2014) is further evidence of altered redox state of *rcd1* mitochondria, and which correlates with metabolomic results and significantly different redox status indicator ratios in this study.

As discussed previously, the most significant differences between Col-0 and *rcd1* in all treatments (L, MV, D) were connected to BCAA and AAA metabolism (**Figures 2** and **3**). In addition, during MV treatment (but not in L or D) the most enhanced pathways between Col-0 and *rcd1* are the TCA cycle and pyruvate metabolism, as well as carbon fixation and sugar metabolism (glycolysis/gluconeogenesis). The differences in carbon fixation (e.g. Calvin cycle) and sugar metabolism is in line with previous results as the photosynthesis is inhibited in Col-0 during MV treatment, but continues in *rcd1* (Shapiguzov et al., 2019). The elevated levels of TCA cycle intermediates (except fumarate) after MV exposure could result from over-active citrate valve, which transports accumulated reducing power out of mitochondria (**Figure 4C**). In plants, depending on the metabolic requirements, redox status and signaling, the fluxes through the mitochondrial TCA cycle can vary (Oliver and McIntosh, 1995; Igamberdiev and Gardeström, 2003; Daloso et al., 2015; Igamberdiev and Bykova, 2018). The increased mitochondrial NAD(P)H levels activate the citrate valve, and excess reducing power is transferred to cytosol or to the other organelles. Partial TCA cycle is reported to be activated in illuminated plants due to the increased mitochondrial redox level, especially in photorespiratory conditions (Igamberdiev and Gardeström, 2003; Igamberdiev and Bykova, 2018), while the normal; full TCA cycle is active during the night. NADH inhibits not only the activity of TCA cycle enzymes isocitrate dehydrogenase (NAD-IDH) and α-ketoglutarate dehydrogenase (α-KGDH), but also (TCA cycle precursor) pyruvate dehydrogenase (PDH) (Oliver and McIntosh, 1995). NAD-IDH is also noncompetitively inhibited by NADPH (McIntosh and Oliver, 1992). The responses of the redox status indicator ratios (**Figure 5**) support the hypothesis of altered redox status in *rcd1* and had a significantly different response to MV exposure in Col-0 and *rcd1*. Citrate/α-KG and isocitrate/α-KG ratios, which describe the status of NAD-IDH, increased significantly in Col-0, but remained in the same level in *rcd1* as in control conditions (light, partial TCA cycle). The α-KG/succinate ratio, which describes the α-KGDH status, decreased in Col-0, but again remained in the same level as in light conditions in *rcd1*. These results indicate that MV exposure does not have the same effect to the mitochondrial redox status in *rcd1* as in wild-type Col-0, even though (organellar specific) increased oxidative stress marker levels indicate ROS production in all cell compartments in both lines during MV exposure (**Figures 4C, D**).

## Conclusion

In addition to photosynthetic metabolism, glycolysis, OPPP and mitochondrial TCA cycle are central nodes of primary metabolism which provide carbon backbones for basal and secondary metabolism, are involved in energy production (ATP) and connect the oxidation of carbon with the reduction of NAD(P) to NAD(P)H. The primary metabolite profile in *rcd1* indicates altered and compartmentalized redox regulation and reorganized energy production. The increased levels of glycolytic and OPPP-derived metabolites as well as altered xanthine/urate ratio indicate altered metabolic regulation and redox imbalance in *rcd1.* We propose that overactive reductive metabolism, activation of energy salvaging pathways and efficient redox transfer between organelles is sufficient to overcome the negative effects of MV-induced chloroplastic oxidative stress in the mutant plants. Accumulation of carbohydrates, BCAAs and AAAs, hydroxy acids and other amino acid catabolites in *rcd1* as well as elevated production of superoxide in *rcd1* in control/light conditions, indicate increased superoxide generation to be connected to altered carbon and nitrogen metabolism and light-dependent reactions in *rcd1*, yet no induction of any of the plastidic oxidative stress markers were detected. The localization of increased superoxide generation as well as organ specific enzyme activities of both redox regulated and enzymes connected to ROS production in the mutant plants needs further studies. Based on previous findings (Shapiguzov et al., 2019) and metabolite features of *rcd1* in our study, we suggest that RCD1 coordinates energy metabolism by negatively regulating reductive metabolism.

## Data Availability Statement

All datasets generated for this study are included in the article/**Supplementary Material**.

## Author Contributions

AS provided all the plant material for starch and metabolite analysis, and performed all the ROS production measurements and stainings. NS and JL performed all of the metabolite and statistical analyses. NS, JL, AS, and MK designed the experiments together. NS, JL, and MK interpreted the results and wrote the manuscript. All authors (NS, JL, AS, MK, JK) were involved in conceptualization and commented the manuscript.

## Conflict of Interest

The authors declare that the research was conducted in the absence of any commercial or financial relationships that could be construed as a potential conflict of interest.
