# Supplementary material for: Primary Metabolite Responses to Oxidative Stress in Early-Senescing and Paraquat Resistant Arabidopsis thaliana rcd1 (Radical-Induced Cell Death1)
Source: Front Plant Sci. 2020 Feb 28;11:194. doi: 10.3389/fpls.2020.00194 (PMC7059619; doi:10.3389/fpls.2020.00194)
Supplement: Supplementary file 1 [file DataSheet_1.pdf]

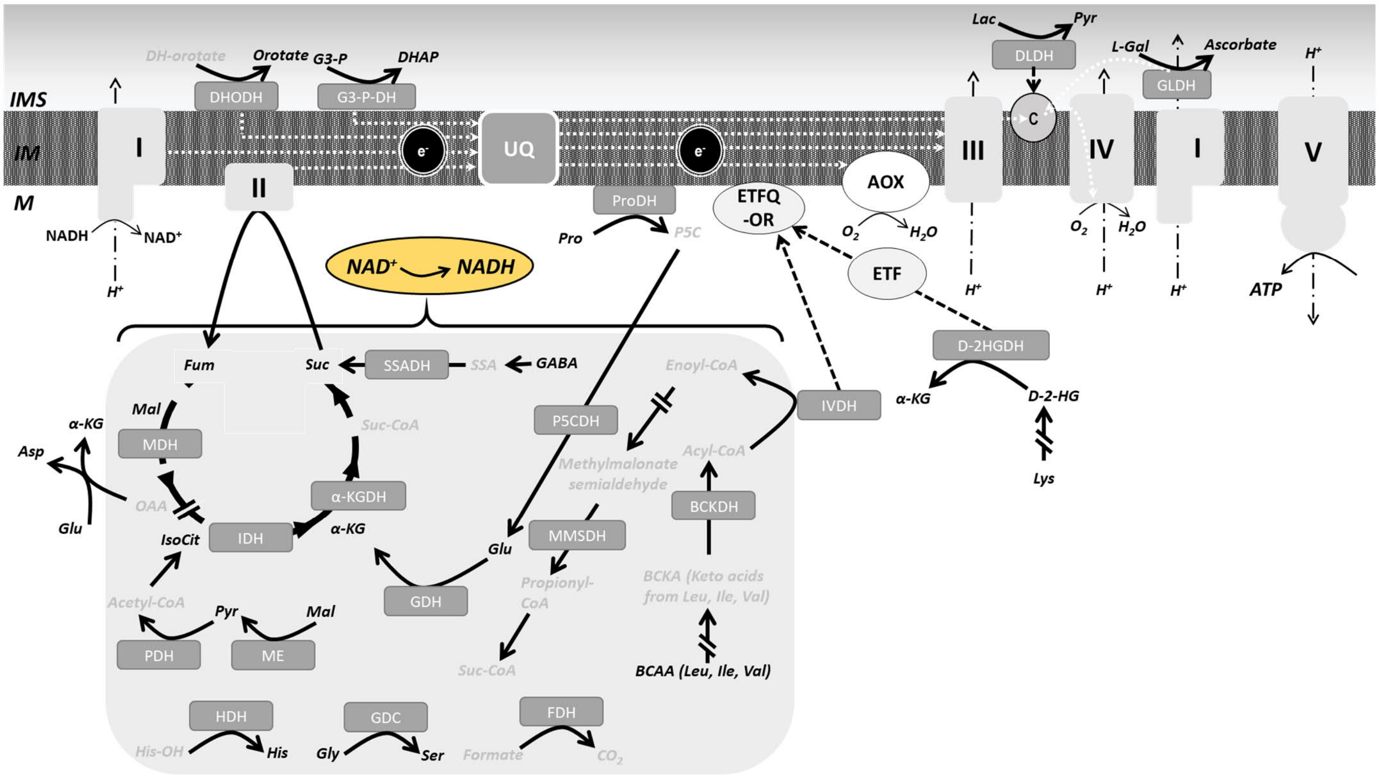

Supplementary Figure S1. Schematic illustration of mitochondrial dehydrogenases and NADH generation based on Schertl and Braun (2014). Redox status indicator ratios (precursor/product) in **Fig. 5** were calculated for all these enzymes (**Supplementary Table S1**), if metabolite data (black) were available, light gray metabolites = not detected.

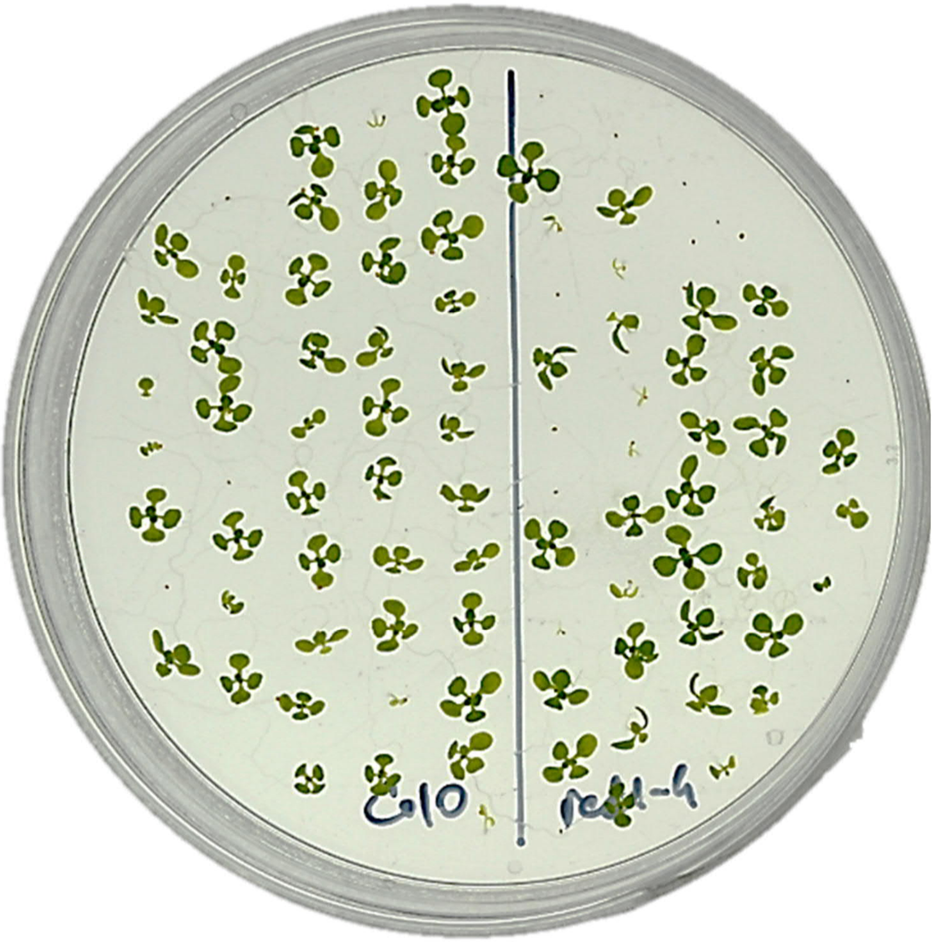

Supplementary Figure S2. Two week old *Arabidopsis thaliana* Col-0 and *rcd1* seedlings grown on agar plate, no visual differences between phenotypes. Left side Col-0, right side *rcd1* in control conditions.

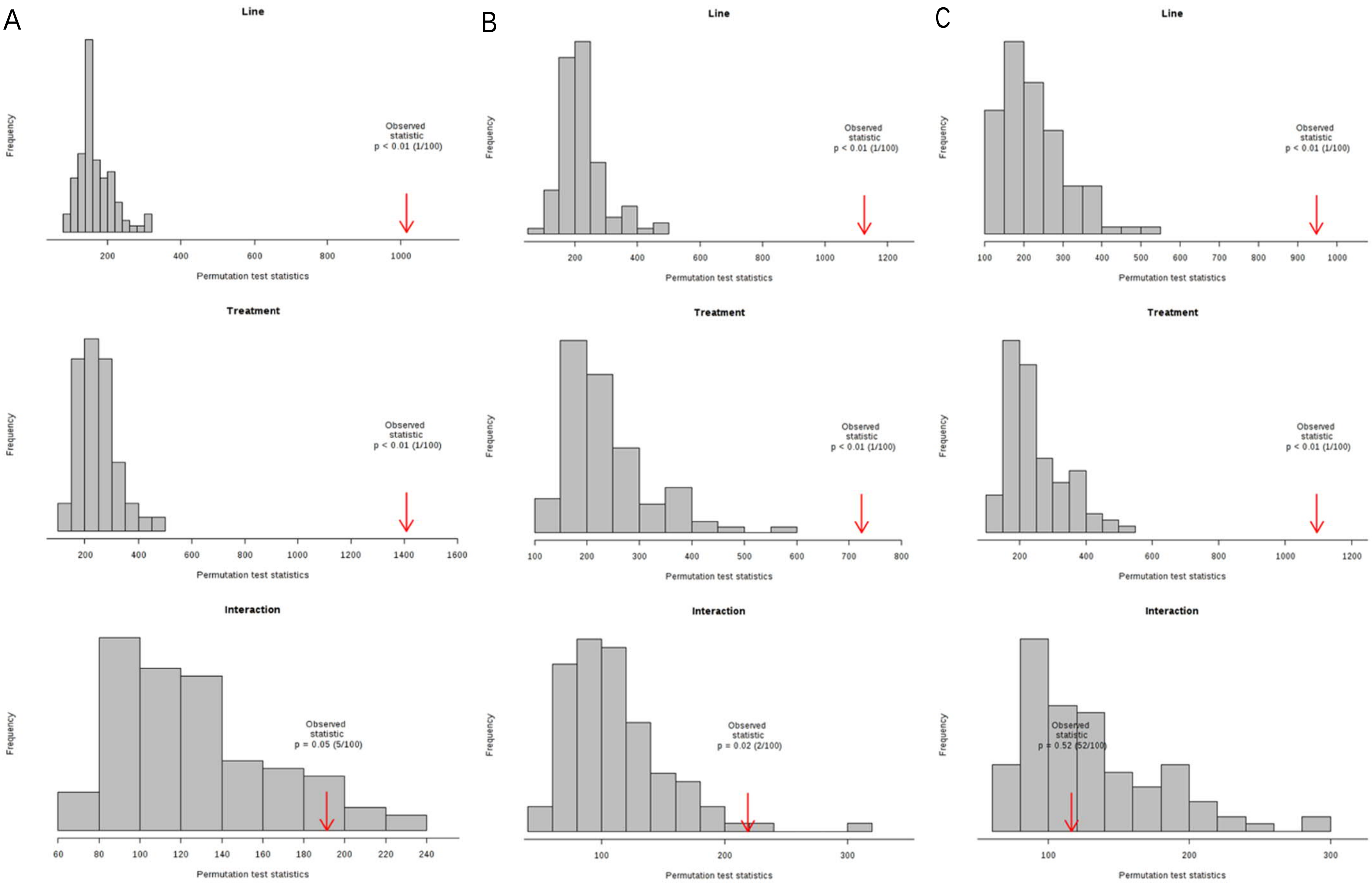

Supplementary Figure S3. ASCA (ANOVA-simultaneous component analysis) was performed with 100 permutations to test the effects of plant line, treatment and their interaction (line × treatment) on the overall variation in the metabolite data (MetaboAnalyst). The effects of line (p-value <0.01), treatment (p-value <0.01), and interaction term (p-value=0.05) were significant (A). The metabolite results were interpreted in the context of all three experimental conditions. ASCA was also performed separately for data sets including L and MV (B), or L and D samples (C).

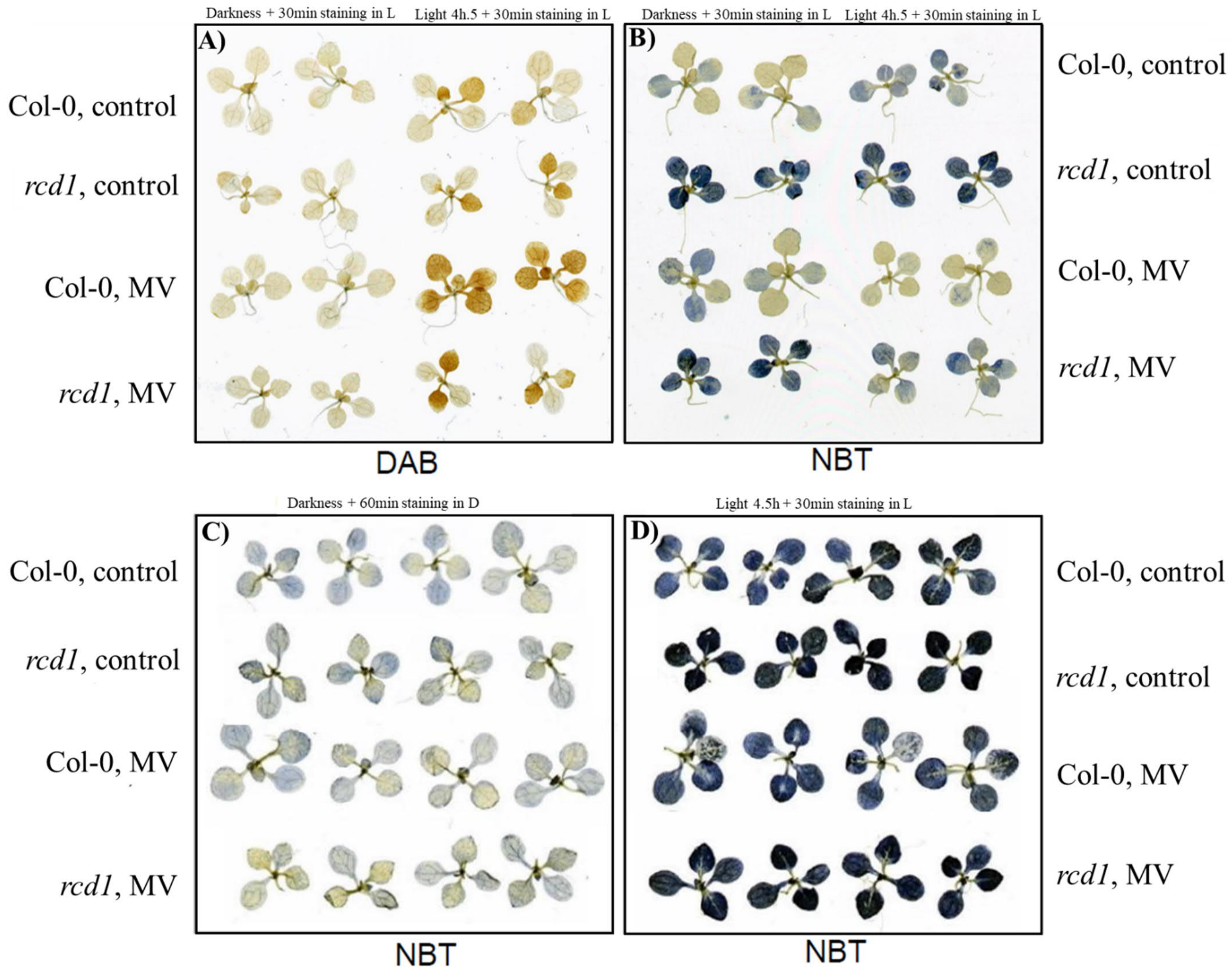

Supplementary Figure S4. Histochemical staining of ROS. **A)** DAB and **B)** NBT staining of two-week-old Col-0 and *rcd1* seedlings with or without MV exposure to detect hydrogen peroxide (DAB) and superoxide production (NBT), staining in 30 min growth light (L). NBT staining in two different conditions; **C)** 60 min in darkness (D) and **D)** 30 min in growth light (L) to illustrate the effect of light to ROS production in Col-0 and *rcd1*. "Darkness" representing 12h (night)+4h continued darkness and "Light" 12h dark (night) + 4.5h light with/without MV, after 2 weeks of 12/12h photoperiod as described in "Plant material" in section Materials and Methods.

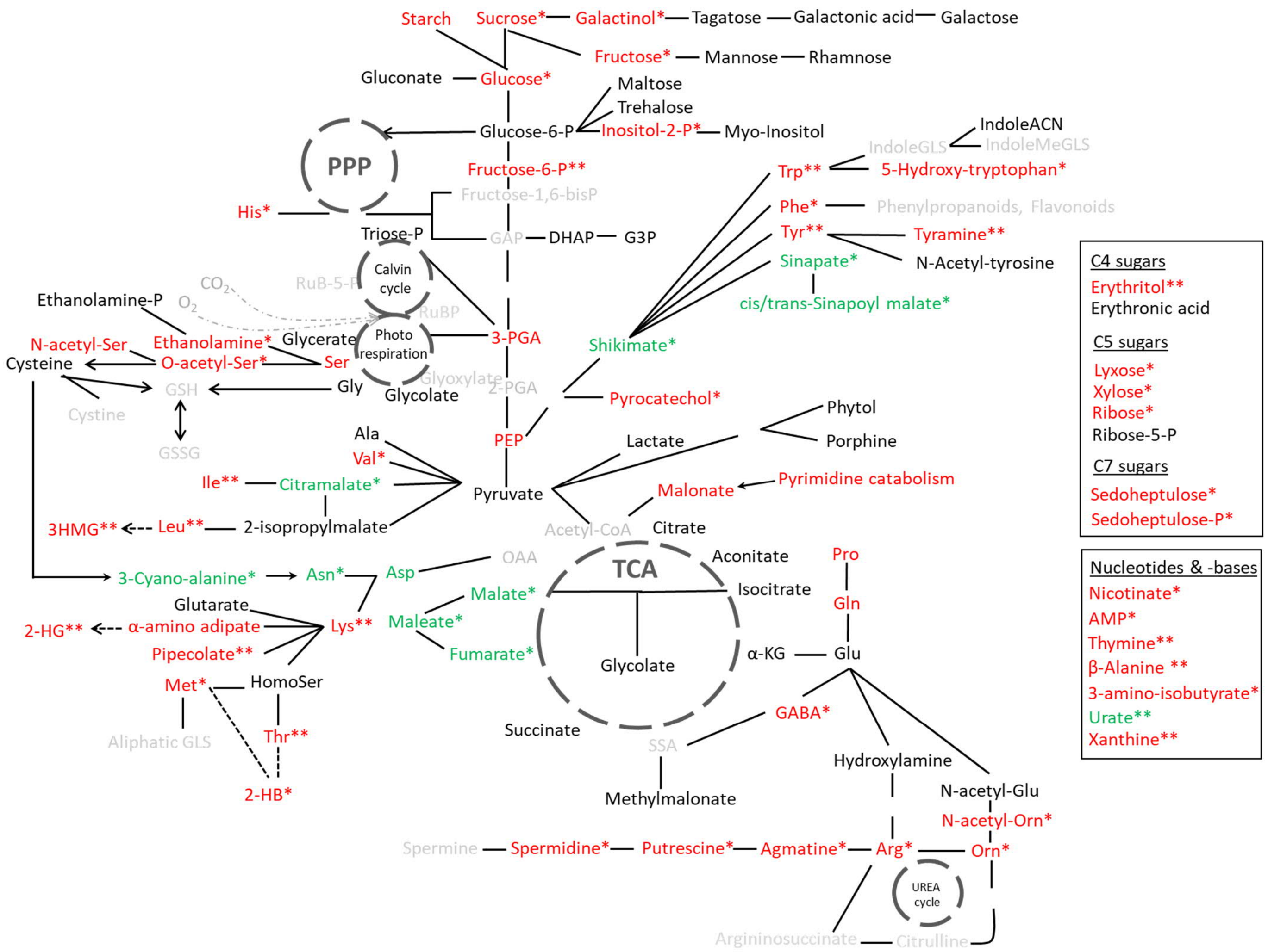

Supplementary Figure S5. Simplified visualization of central carbon and nitrogen pathways in Col-0 vs *rcd1* in control conditions (light). Red indicates significant increase and green significant decrease in *rcd1* compared to Col-0. Grey, not detected (n.d.) and black, no significant difference. The effect of plant line on metabolite levels was tested with T-test (p-value <0.05*, <0.01**, IBM SPSS Statistics 22).
